# How the risky features of previous selection affect subsequent decision-making: evidence from behavioral and fMRI measures

**DOI:** 10.3389/fnins.2015.00364

**Published:** 2015-10-06

**Authors:** Guangheng Dong, Yifen Zhang, Jiaojing Xu, Xiao Lin, Xiaoxia Du

**Affiliations:** ^1^Department of Psychology, Zhejiang Normal UniversityJinhua, China; ^2^Peking-Tsinghua Centre for Life Science, Peking UniversityBeijing, China; ^3^Shanghai Key Laboratory of Magnetic Resonance, Department of Physics, East China Normal UniversityShanghai, China

**Keywords:** decision making, prior risk experience, risk-taking, fMRI

## Abstract

Human decision making is rarely conducted in temporal isolation. It is often biased and affected by environmental variables, particularly prior selections. In this study, we used a task that simulates a real gambling process to explore the effect of the risky features of a previous selection on subsequent decision making. Compared with decision making after an advantageous risk-taking situation (Risk_Adv), that after a disadvantageous risk-taking situation (Risk_Disadv) is associated with a longer response time (RT, the time spent in making decisions) and higher brain activations in the caudate and the dorsolateral prefrontal cortex (DLPFC). Compared with decisions after Risk_Adv, those after Risk_Disadv in loss trials are associated with higher brain activations in the left superior temporal gyrus (STG) and the precuneus. Brain activity and relevant RTs significantly correlated. Overall, people who experience disadvantageous risk-taking selections tend to focus on current decision making and engage cognitive endeavors in value evaluation and in the regulation of their risk-taking behaviors during decision making.

## Introduction

Decision making requires the ability to select from competing actions that are associated with varying levels of risk and reward. Human decision making is rarely conducted in temporal isolation. Current choices are always affected by environmental variables and often evaluated depending on the outcomes preceded by choices (Ernst and Paulus, 2005; Xue et al., 2011; Drugowitsch et al., 2012). Cumulative evidence has shown that human decision making is affected by previous selections even when participants are informed that trials are independent and outcomes are random (Cohen and Aston-Jones, 2005; Hecht et al., 2010; Dong et al., 2014a,b).

Various neuroscience approaches have been recently used to identify the neural mechanisms underlying risky decision making (Pabst et al., 2013; Lewis et al., 2014). Certain brain regions, such as the inferior frontal gyrus (Cazzell et al., 2012; Fukunaga et al., 2012; Rushworth et al., 2012; Sheth et al., 2012), can signal subjective risk and facilitate the formation of subjective feelings during decision making (Christopoulos et al., 2009; Craig, 2009; Cazzell et al., 2012; Fukunaga et al., 2012). The anterior cingulate cortex has been associated with error monitoring, conflict detection, and performance monitoring in decision making (Holroyd and Coles, 2002; van Veen et al., 2004; Platt and Huettel, 2008). Other works have identified risk-related brain regions, such as the lateral orbitofrontal cortex, the insula (Critchley et al., 2001; Kuhnen and Knutson, 2005), and the caudate (Elliott et al., 2003; Grahn et al., 2009; Foerde and Shohamy, 2011), which are also responsive to monetary gains and/or losses. The dorsolateral prefrontal cortex (DLPFC) and the anterior insula are more active when selecting risky vs. safe options (Paulus et al., 2003; Kuhnen and Knutson, 2005; Schonberg et al., 2011).

Two aspects affect human decision making. One includes current decisions from the outcomes (win/loss) of prior selections. For example, participants who lose in a gamble are more risky than those who win (Xue et al., 2010, 2011). Shiv et al. (2005) studied the behavior of healthy controls and brain-damaged control patients who participated in an investment game where gains and losses are determined by a coin toss and found that these individuals tend to quit after losing. The other is the risky experience in a previous decision process. The avoidance of risky behaviors, particularly those related to the experience of loss, is a central feature of decision making (Rothman and Salovey, 1997). Xue et al. (2010) found that the insula activates representations of homeostatic states associated with the experience of risk, which consequently affects subsequent decisions.

The neuroscience of decision making under risk has focused on identifying brain systems that shape behavior toward or against particular choices (Hsu et al., 2005; Huettel et al., 2006; Platt and Huettel, 2008; San Martin et al., 2014). These studies typically ask participants to choose between a safer, lower-value option, and a riskier, higher-value option (Coricelli et al., 2005; De Martino et al., 2006; Tom et al., 2007; Venkatraman et al., 2009). The presence of “risk” indicates higher rates to lose but offers an opportunity to win a large amount (higher reward). Studies have primarily focused on the brain features of decision making and rarely on the influence of a previous selection and their results on current decision making. In the present study, we explored the effect of risky features in a previous selection on subsequent decision making at the whole-brain level by using neuroimaging techniques in conjunction with a risky decision-making task that simulates a gambling process. The behavioral and brain reactions of the participants were measured and compared under different conditions. We first divided all trials into “advantageous risk-taking (Risk_Adv)” and “disadvantageous risk-taking (Risk_Disadv)” situations. We further divided each condition into after-win and after-loss on the basis of the outcomes of previous selections to explore the behavioral responses and cognitive mechanisms under this process. Compared with people who won in a previous decision, people who lost in a previous decision may become frustrated and cautious in the subsequent selection; hence, the latter group may need a longer time for decision making. The caudate is important in the reward circuits and is reportedly involved in anticipation and performance-related feedback (Seger and Cincotta, 2005; Tricomi et al., 2006). In the current study, the win/loss will activate the reward/punishment experience of the participants. We hypothesized that Risk_DisAdv recruits higher caudate activations because it offers an opportunity to win a large amount. During risky decision making, executive inhibition controls impulse. The anterior cingulate cortex (Holroyd and Coles, 2002; Platt and Huettel, 2008) and the DLPFC (Paulus et al., 2003; Schonberg et al., 2011) are associated with executive control in decision making. Thus, we hypothesized that executive control-related brain regions are involved in the process. In addition, we aimed to find correlations between brain changes and relevant behavioral performances because of two reasons. First, different processes may be operating in parallel during the task. Interpretation is enhanced if multiple brain regions that show changes can be linked to separable behavioral effects. Second, individual differences during a task may not be understood without measuring behavioral outcomes.

## Methods and materials

### Participant selections

Twenty-two healthy young adults (age: 22.2 ± 1.8 years) participated in this study. They provided written informed consent, which was approved by The Human Investigations Committee at Zhejiang Normal University. None of them reported current Axis I disorders as assessed, using structured psychiatric interviews (M.I.N.I.) (Lecrubier et al., 1997) by an experienced psychiatrist. All subjects are right handed and have not suffered head injuries with lost of consciousness during their lifetime.

### Task and procedure

**Figure 1:**
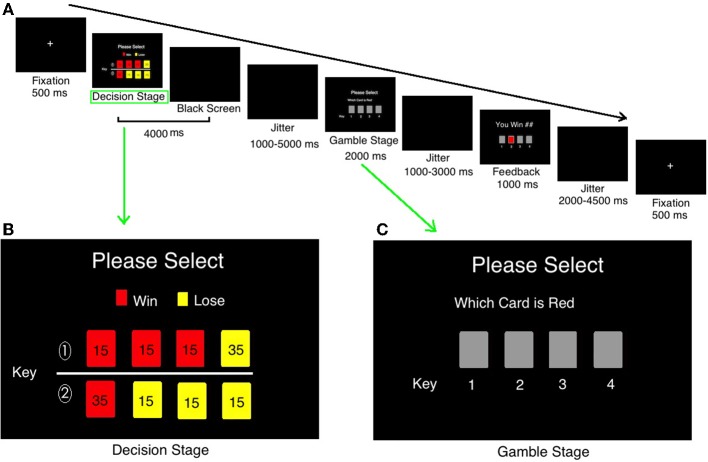
**The timeline of one trial in current study**. **(A)** The timeline of one trial in current study; **(B)** the detailed information in “Decision” stage; **(C)** the detailed information in “Gamble” stage.

The task used a rapid event-related design. This task consisted of 80 trials. Each trial was divided into three stages: Decision stage, Gamble stage, and Feedback stage. Figure 1A shows the event sequence of each trial during the task. A white cross was presented at the center of a black screen for 500 ms to cue the beginning of a new trial. During the decision stage, participants were asked to choose between two risky options (see details on decision stage in Figure 1B). This selection process would last for 4000 ms at most or will disappear once the participant made a decision. After a varied period of delay (mean 3000 ms, ranging from 1000 to 5000 ms), there came the gamble stage (Figure 1C). During the gamble stage, participants would see the backs of 4 cards and be asked to guess which one was red and indicate their responses by a button press within 2000 ms (the order of the cards during the gamble state was randomized). If they missed, they would lose 15 Chinese Yuan (about $2.5 USD). After the response and a delay ranging from 1000 to 3000 ms (mean 2000 ms), the selected card would turn over and showed the feedback to inform participants of the outcome, which would be presented for a period of 1000 ms. Participants would win/lose the amount according to the card color and the number on the card. The jitter would be presented after that for 1000–3000 ms. The next trial would begin after a jittered delay (mean 3000 ms, ranging from 2000 to 4500 ms). The whole experiment was presented by E-prime software (Psychology Software Tools, Inc.).

#### Decision stage

During the decision stage, two lines of cards (each line consists of 4 cards) were presented on the computer screen (see Figure 1B), with the red color and the amount on it suggesting winning an amount, and the yellow suggesting losing some amount. The cards were shown in colors to indicate the results (red, win; yellow, loss), win/loss rates (the proportions of different color cards), and win/loss amount (the number on cards).

### Advantages/disadvantageous risk-taking conditions

The probability and magnitude of the gain/loss were manipulated to create advantageous/disadvantageous risk-taking selections.

In Figure 1B, the first line is advantageous risk-taking condition [(45 * 0.75) + (−35 * 0.25) = 25 Yuan]. The advantageous risk-taking means the sum of numbers on red cards (win) are larger than that in yellow ones (loss). It suggests that although participants have opportunities to lose a big amount, they are more likely to win money in the long run. On the contrary, the second line in Figure 1B is disadvantageous risk-taking condition: the gross of red card (win) is smaller than that of all yellow cards [(35 * 0.25) + (−45 * 0.75) = −25 Yuan]. It suggests that although participants have opportunities to win a big amount, however, they are more likely to lose money in the long run. Participants were practiced using the same task before formal scan.

Participants were told they had 80 times to win some money. And each trial was presented randomly throughout the task. Each participant was provided with 200 Chinese Yuan as the initial balance before the task, and was explicitly informed that he or she would receive the entire balance in cash at the end of the task. The win/loss rates of their selections in different conditions were pseudo-randomized, which was to balance the trial numbers in different conditions. Subjects were practiced the same task for 15 trials before scan, which is to let them familiar with the task. The risky features of different conditions were introduced by the researchers before experiment.

We first defined two different task conditions based on the risky features of their previous selections: (1) decision making after advantageous risk-taking trials (Risk_Adv); (2) decision making after disadvantageous risk-taking trials (Risk_DisAdv). Second, we further divided each of these conditions into two different ones, according to the outcomes of their previous selections: (1) decision making after advantageous/disadvantageous risk-taking and win trials (Risk_Adv/DisAdv_Win); (2) decision making after advantageous/disadvantageous risk-taking and loss trials (Risk_Adv/DisAdv_Loss).

Participants who chose the same selections for more than 50 percent of all trials (they might have selection bias) or chose the same selections for more than 10 times (they might be lack of motivation to perform properly) were excluded from further analysis. Participants who had less than 10 trials in one of these four conditions were excluded from further analysis to keep the statistical power. In this study, we only focused on how the previous selections and its outcomes would affect current decision-making process.

### Image acquisition and pre-processing

The image acquisition parameters have been described previously (Dong et al., 2013) and are as follows. Structural images covering the whole brain were collected, via a T1-weighted three-dimensional spoiled gradient-recalled sequence [176 slices, *TR* = 1700 ms, echo time (*TE*) = 3.93 ms, slice thickness = 1.0 mm, skip = 0 mm, flip angle = 15°, inversion time = 1100 ms, field of view (FOV) = 240 × 240 mm, in-plane resolution = 256 × 256). Functional MRI was performed on a 3T scanner (Siemens Trio) with a gradient-echo EPI T2 sensitive pulse sequence in 33 slices (interleaved sequence, 3 mm thickness, *TR* = 2000 ms, *TE* = 30 ms, flip angle = 90°, field of view = 220 × 220 mm^2^, matrix = 64 × 64). Stimuli were presented via *Invivo* synchronous system (*Invivo* Company, www.invivocorp.com/) through a screen in the head coil, enabling participants to view the stimuli. A total of 630 volumes were acquired for each participant during the 1260 s of task performance.

### First-level regression analysis

The functional data were analyzed using SPM5 and Neuroelf (http://neuroelf.net) as described previously (DeVito et al., 2012; Dong et al., 2013; Krishnan-Sarin et al., 2013). Images were slice-timed, corrected, reoriented (manually), and realigned to the first volume. T1-co-registered volumes were then normalized to an MNI template and spatially smoothed with a 6 mm FWHM Gaussian kernel. In this study, we only paid attention to the risk selection process (decision stage). The next stimulus (results of the decision) was analyzed in other studies.

A general linear model (GLM) was applied to identify blood oxygen level dependence (BOLD) activation in relation to separating event types. The six head-movement parameters derived from the realignment stage were included as covariates of no interest. In addition, reward history (cumulated win-lose amount before the present trial), and response history (stay/switch to previous selections) were included as parameters in the model to eliminate their potential influence to the results. For these conditions, the duration is 4000 ms. There are 11 predictors in the model (2 interested conditions: decision making after advantageous/disadvantageous selections; and variables of no interest (6 head movement parameters; 2 of the outcomes of previous selection conditions: win, lose; 1 reward history condition (the cumulated amount of the win/lose balance). Further analysis includes 4 interested conditions: decision making after Risk_DisAdv Win/lose; decision making after Risk_Adv Win/lose, and 9 other predictors as described above. All valid trials were included in the analysis. GLM was independently applied to each voxel to identify voxels that were significantly activated for the different events of each condition.

### Second-level group analysis

Second level analysis treated inter-subject variability as a random effect. Primarily, we determined to take voxels to show a main effect in different conditions. Second, we tested for voxels that showed higher or lower activity in all contrasts of interest. We first identified clusters of contiguously significant voxels at an uncorrected threshold *p* < 0.01, as also used for displaying purposes in the figures. We then tested these clusters for cluster-level FWE correction *p* < 0.01 and the AlphaSim estimation indicated that clusters with 42 contiguous voxels would achieve an effective FWE threshold *p* < 0.01. The smoothing kernel used during simulating false-positive (noise) maps with AlphaSim was 6.0 mm, and was estimated from the residual fields of the contrast maps entered into the one-sample *t*-test. The formula used to compute the smoothness was that used in FSL (see http://www.fmrib.ox.ac.uk/analysis/techrep/tr00df1/tr00df1/node6.html for more information).

### Correlation analysis

We first compared the brain activations between “Risk_DisAdv” and “Risk_Adv” and then took the surviving clusters as ROIs for further analyses. For each ROI, a representative beta value was obtained by averaging the signal of all the voxels within the ROI (We took the survived clusters as ROIs for further analysis. The beta values for each subject were abstracted from grouped level mask into individual space). We calculated correlations to support our hypothesis: correlations between the brain activity (beta value) in caudate in Risk_DisAdv/Risk_DisAdv_Win and relevant response time (RT); correlation between brain activity (beta value) in precuneus in Risk_DisAdv_Lose and relevant RT.

## Results

### Behavioral performance

**Figure 2:**
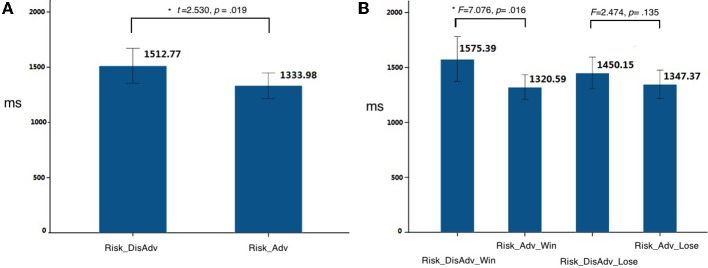
**Behavioral performances in current study**. **(A)** RT between Risk_DisAdv and Risk_Adv in all trials; **(B)** RT between Risk_DisAdv and Risk_Adv in win/lose trials.

The decision-making after Risk_DisAdv showed significant longer response time (RT, the time they spend in making decisions in decision stage in current trial) [*t*_(21)_ = 2.530, *p* = 0.019, *d* = 0.73 than that after Risk_Adv] (Figure 2A). Further analysis separating these conditions into win and loss, according to the outcome of their previous selections, showed that the Risk_DisAdv_Win was associated with longer RT than that after Risk_Adv_Win [*F*_(3, 19)_ = 7.076, *p* = 0.016, $\eta_{p}^{2}=0.05$]; the Risk_DisAdv_Lose showed longer RT than Risk_Adv_Lose [*F*_(3, 19)_ = 2.474, *p* = 0.135, $\eta_{p}^{2}=0.12$], although it does not reach statistical significant (Figure 2B). No interactions were found between advantageous/disadvantageous and win/lose of previous selections in current study [*F*_(1, 21)_ = 0.243, *p* = 0.622]. The repeating rates (subjects selected the same risky feature as their previous selections) in Risk_DisAdv (0.35 ± 0.13) were significantly lower than that in Risk_Adv (0.62 ± 0.17) [*t*_(21)_ = 2.68, *p* < 0.01, *d* = 0.69]. The stay/switch rates after advantageous and win decisions are 78%: 22% [*F*_(3, 19)_ = 6.86, *p* < 0.001, $\eta_{p}^{2}=0.04$]; the rates are 67%: 33% [*F*_(3, 19)_ = 4.69, *p* < 0.001, $\eta_{p}^{2}=0.07$] in advantageous and lose choices. In addition, the stay/switch rates in disadvantageous and win situations are 52%: 48% [*F*_(3, 19)_ = 1.22, *p* > 0.05, $\eta_{p}^{2}=0.012$]; and it is 34%: 66% in dis-advantageous and lose choices [*F*_(3, 19)_ = 5.04, *p* < 0.001, $\eta_{p}^{2}=0.05$].

### Imaging results

#### Risk_DisAdv > Risk_Adv in all trials

**Figure 3:**
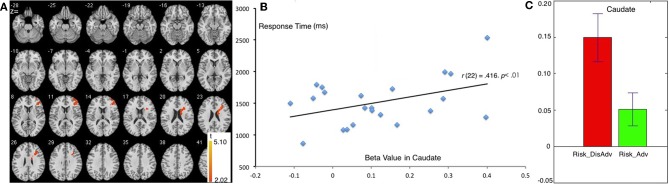
**Comparison between Risk_DisAdv and Risk_Adv in all trials**. **(A)** Imaging results show the Risk_DisAdv elicited higher brain activation in right caudate and DLPFC; **(B)** Correlation between RT and brain activities in Caudate in Risk_DisAdv; **(C)** Beta figures in caudate in Risk_DisAdv and Risk_Adv.

Compared with the Risk_Adv, the Risk_DisAdv showed higher brain activation in the right caudate and right DLPFC (Table 1, Figure 3A). Significant positive correlation was found between the brain activity in Caudate in Risk_DisAdv and relevant RT (Figure 3B). Beta figure showed that Risk_DisAdv elicited higher brain activity in the Caudate (Figure 3C).

**Table 1:** **Regional brain activity changes in different comparisons**.

**x, y, z^a^**	**Hemisphere**	**Peak intensity**	**Cluster size^b^**	**Region^c^**	**Brodmann's area**
**RISK_DISADV** > **RISK_ADV**					
15, −6, 24	R	4.289	97	Caudate	
42, 36, 12	R	3.892	67	DLPFC	46
**RISK_DISADV_WIN** > **RISK_ADV_WIN**					
15, −6, 21	R	5.029	142	Caudate	
45, 39, 12	R	4.315	94	DLPFC	46
**RISK_DISADV_LOSE** > **RISK_ADV_LOSE**					
−36, −48, 12	L	4.304	139	Superior temporal gyrus	22
24, −33, 66	R	3.908	89	Precuneus	4

a *Peak MNI Coordinates*.

b Number of voxels. We first identified clusters of contiguously significant voxels at an uncorrected threshold p < 0.01, as also used for display purposes in the figures. We then tested these clusters for cluster-level FWE correction p < 0.01 and the AlphaSim estimation indicated that clusters with 42 contiguous voxels would achieve an effective FWE threshold p < 0.01. Voxel size = 3 ^*^ 3 ^*^ 3.

c *The brain regions were referenced to the software Xjview (http://www.alivelearn.net/xjview8) and double checked with atlas*.

#### Risk_Adv_Win > Risk_DisAdv_Win

**Figure 4:**
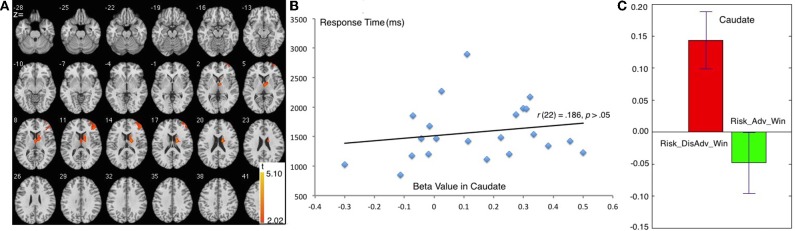
**Comparison between Risk_DisAdv and Risk_Adv in win trials**. **(A)** Imaging results show the Risk_DisAdv_Win elicited higher brain activation in right caudate and DLPFC; **(B)** Correlation between RT and brain activities in Caudate in Risk_DisAdv_Win; **(C)** Beta figures in Caudate in Risk_DisAdv_Win and Risk_Adv_Win.

The comparison between Risk_Adv_Win and Risk_DisAdv_Win showed great similarity to the comparison between Risk_Adv and Risk_DisAdv. During the process of Risk_DisAdv_Win, relative to Risk_Adv_Win, greater BOLD signal was observed in caudate, and right DLPFC (Table 1, Figure 4A). Marginally significant positive correlation was found between the brain activities in caudate in Risk_DisAdv_Win and relevant RT (Figure 4B). The beta figure showed that the Risk_DisAdv_Win elicited higher brain activations in caudate (Figure 4C).

#### Risk_Adv > Risk_DisAdv after lose trials

**Figure 5:**
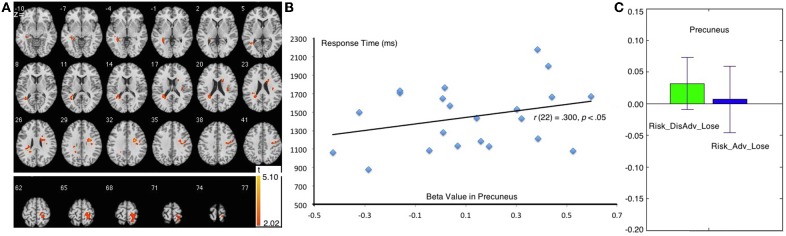
**Comparison between Risk_DisAdv and Risk_Adv in lose trials**. **(A)** Imaging results show the Risk_DisAdv_Win elicited higher brain activation in left STG and Precuneus; **(B)** Correlation between RT and brain activities in precuneus in Risk_DisAdv_Lose; **(C)** Beta figures in precuneus in Risk_DisAdv_Lose and Risk_Adv_Lose.

The Risk_DisAdv_Lose, relative to Risk_Adv_Lose, showed increased BOLD signal in the left superior temporal gyrus, and right precuneus (Table 1, Figure 5A). Significant positive correlation was found between the brain activities in precuneus in Risk_DisAdv_Lose and relevant RT (Figure 5B). The beta figure showed that the Risk_DisAdv_Lose elicited higher brain activations in the Precuneus (Figure 5C).

## Discussion

Using a task that simulates real-life gambling, we found that the risky features of previous selections can affect current decision making. These effects can be observed in behavioral and brain activities.

### Risk_DisAdv > Risk_Adv in all trials

The comparison between Risk_DisAdv and Risk_Adv shows that the risky features of previous decisions can affect current decision making. Neuroimaging results show that Risk_DisAdv is associated with high brain activation in the right caudate and the right DLPFC, which supports our hypothesis. Neuroimaging and anatomical studies show that the caudate is fundamental to the selection of behaviors on the basis of the changing values of goals and knowledge of which actions lead to what outcomes (Grahn et al., 2009; Foerde and Shohamy, 2011) and whether or not to trust another person when money is at stake (Elliott et al., 2003). The caudate is also reportedly involved in anticipation and performance-related feedback (Seger and Cincotta, 2005; Tricomi et al., 2006). A recent study that used a guessing task with monetary outcomes has reported that the caudate is recruited only when participants believe the existence of contingencies between their actions and the subsequent results (received a reward or punishment) (Tricomi and Fiez, 2008). These results suggest that the caudate performs a role in evaluating values during decision making. In this study, Risk_DisAdv shows higher brain activations in the caudate than Risk_Adv. The beta figure of caudate activation demonstrates that Risk_DisAdv elicits higher activations than Risk_Adv. In behavioral performance, the RT in decisions after Risk_DisAdv is considerably longer than that in decisions after Risk_Adv. RT and brain activations significantly positively correlate under Risk_DisAdv condition. This finding indicates that brain activations increase as the time needed in making decisions is prolonged. We therefore conclude that people engage more endeavors in value evaluation during decision making after Risk_DisAdv than after Risk_Adv.

As hypothesized, the right DLPFC is highly activated in decision making after Risk_DisAdv trials. The DLPFC is involved in risky decision making (Greene et al., 2001). In addition, the DLPFC is activated when costs and benefits of alternative choices are of interest (Duncan and Owen, 2000). Similarly, the DLPFC evokes a preference toward the most equitable option and suppresses the temptation to maximize personal gain when options for choosing alternatives are present (Knoch and Fehr, 2007). fMRI studies suggest that the right DLPFC regulates risk-taking behaviors (Ernst and Paulus, 2005). The transient disruption of the right DLPFC increases risky decision making in a gambling task (Knoch and Fehr, 2007). These results support the suggestion that the DLPFC regulates risk-taking behaviors during decision making. In this study, the decisions after Risk_DisAdv show higher brain activation in the DLPFC than those after Risk_Adv. Thus, we conclude that people engage more cognitive endeavors in regulating their risk-taking behavior in decisions after Risk_DisAdv trials than after Risk_Adv trials. The lower repeating rates in Risk_DisAdv than in Risk_Adv also support the conclusion that people regulate their risk-taking behaviors.

### Risk_DisAdv > Risk_Adv after win trials

Further comparison between decision making after Risk_Adv and Risk_DisAdv after win trials exhibits a marked similarity to Risk_DisAdv > Risk_Adv in all trials. Neuroimaging results reveal that Risk_DisAdv_Win shows higher activation than Risk_Adv_Win in the caudate and the right DLPFC. The beta figure shows that Risk_DisAdv shows higher brain activations under_Win conditions. These results will not be discussed in this study because of the great similarities.

### Risk_DisAdv > Risk_Adv in decisions after lose trials

Compared with decisions after Risk_Adv_Loss, those after Risk_DisAdv_Loss are associated with higher brain activations in the left superior temporal gyrus (STG) and the precuneus. The STG is involved in the perception of negative emotions (Bigler et al., 2007; Radua et al., 2010). The negative trend of activity in the STG reflects a redistribution of resources from areas implicated in cognitive processing to those directly involved in emotion processing (Plewnia et al., 2007). In this study, the higher activity in the STG in decision making suggests that people experience more negative emotions after Risk_DisAdv_Loss than after Risk_Adv_Loss. This result also supports the conclusion that Risk_DisAdv_Loss elicits a higher negative experience during this process. The higher negative emotion after Risk_DisAdv_Loss than after Risk_Adv_Loss is easily understandable because of the money loss involved in the former situation. Money is also lost after Risk_Adv_Loss, but the amount lost is considerably smaller in this situation than in Risk_DisAdv_Loss.

The right precuneus is another brain area that survived after the comparison between Risk_DisAdv_Loss and Risk_Adv_Loss. Precuneus activities reflect increased visual attention due to difficult task demands (Barber and Carter, 2005; Remijnse et al., 2013). Astafiev (Astafiev et al., 2003) found that the precuneus is more active in challenging tasks than in simple tasks. These results suggest that the activity of the precuneus increases with attentional demands for stimulus detection. In this study, the higher brain activation in Risk_DisAdv_Loss than in Risk_Adv_Loss suggests that great attention is engaged in current selections. In behavioral performance, the trial RT after Risk_DisAdv_Loss is longer than that after Risk_Adv_Loss, although the difference is not statistically significant. The positive correlation between brain activations in Risk_DisAdv_Loss and relevant RT suggests that longer time engaged in decision making translates to higher observable precuneus activations.

We therefore conclude that people engage greater attention in their current decisions after Risk_DisAdv_Loss than after Risk_Adv_Loss.

## Conclusions

People engage greater attention in current decision making after Risk_DisAdv than after Risk_Adv. Moreover, people engage more cognitive endeavors in value evaluation and in the regulation of their risk-taking behaviors after Risk_DisAdv than after Risk_Adv.

## Author contributions

GD designed the research and wrote the manuscript, YZ and XL contributed in data collecting, data analyzing and figure preparing. JX contributed in manuscript preparing. XD contributed in data collecting and preprocessing.

## Funding

The funders had no role in study design, data collection, and analysis, decision to publish, or preparation of the manuscript. The contents of the manuscript do not necessarily reflect the views of the funding agencies.

### Conflict of interest statement

The authors declare that the research was conducted in the absence of any commercial or financial relationships that could be construed as a potential conflict of interest.

## References

[B1] Astafiev S. V. Shulman G. L. Stanley C. M. Snyder A. Z. Van Essen D. C. Corbetta M. (2003). Functional organization of human intraparietal and frontal cortex for attending, looking, and pointing. J. Neurosci. 23, 4689–4699. 12805308 10.1523/JNEUROSCI.23-11-04689.2003 PMC6740811

[B2] Barber A. D. Carter C. S. (2005). Cognitive control involved in overcoming prepotent response tendencies and switching between tasks. Cereb. Cortex 15, 899–912. 10.1093/cercor/bhh189 15459082

[B3] Bigler E. D. Mortensen S. Neeley E. S. Ozonoff S. Krasny L. Johnson M.. (2007). Superior temporal gyrus, language function, and autism. Dev. Neuropsychol. 31, 217–238. 10.1080/87565640701190841 17488217

[B4] Cazzell M. Li L. Lin Z. J. Patel S. J. Liu H. (2012). Comparison of neural correlates of risk decision making between genders: an exploratory fNIRS study of the Balloon Analogue Risk Task (BART). Neuroimage 62, 1896–1911. 10.1016/j.neuroimage.2012.05.030 22634214

[B5] Christopoulos G. I. Tobler P. N. Bossaerts P. Dolan R. J. Schultz W. (2009). Neural correlates of value, risk, and risk aversion contributing to decision making under risk. J. Neurosci. 29, 12574–12583. 10.1523/JNEUROSCI.2614-09.2009 19812332 PMC2794196

[B6] Cohen J. D. Aston-Jones G. (2005). Cognitive neuroscience: decision amid uncertainty. Nature 436, 471–472. 10.1038/436471a 16049461

[B7] Coricelli G. Critchley H. D. Joffily M. O'Doherty J. P. Sirigu A. Dolan R. J. (2005). Regret and its avoidance: a neuroimaging study of choice behavior. Nat. Neurosci. 8, 1255–1262. 10.1038/nn1514 16116457

[B8] Craig A. D. (2009). How do you feel–now? The anterior insula and human awareness. Nat. Rev. Neurosci. 10, 59–70. 10.1038/nrn2555 19096369

[B9] Critchley H. D. Mathias C. J. Dolan R. J. (2001). Neural activity in the human brain relating to uncertainty and arousal during anticipation. Neuron 29, 537–545. 10.1016/S0896-6273(01)00225-2 11239442

[B10] De Martino B. Kumaran D. Seymour B. Dolan R. J. (2006). Frames, biases, and rational decision-making in the human brain. Science 313, 684–687. 10.1126/science.1128356 16888142 PMC2631940

[B11] DeVito E. E. Worhunsky P. D. Carroll K. M. Rounsaville B. J. Kober H. Potenza M. N. (2012). A preliminary study of the neural effects of behavioral therapy for substance use disorders. Drug Alcohol. Depend. 122, 228–235. 10.1016/j.drugalcdep.2011.10.002 22041256 PMC3296894

[B12] Dong G. Hu Y. Lin X. Lu Q. (2013). What makes Internet addicts continue playing online even when faced by severe negative consequences? Possible explanations from an fMRI study. Biol. Psychol. 94, 282–289. 10.1016/j.biopsycho.2013.07.009 23933447

[B13] Dong G. Lin X. Zhou H. Du X. (2014a). Decision-making after continuous wins or losses in a randomized guessing task: implications for how the prior selection results affect subsequent decision-making. Behav. Brain Funct. 10:11. 10.1186/1744-9081-10-11 24708897 PMC4234378

[B14] Dong G. Lin X. Zhou H. Lu Q. (2014b). How the win–lose balance situation affects subsequent decision-making: functional magnetic resonance imaging evidence from a gambling task. Neuroscience 272, 131–140. 10.1016/j.neuroscience.2014.04.058 24814016

[B15] Drugowitsch J. Moreno-Bote R. Churchland A. K. Shadlen M. N. Pouget A. (2012). The cost of accumulating evidence in perceptual decision making. J. Neurosci. 32, 3612–3628. 10.1523/JNEUROSCI.4010-11.2012 22423085 PMC3329788

[B16] Duncan J. Owen A. M. (2000). Common regions of the human frontal lobe recruited by diverse cognitive demands. Trends Neurosci. 23, 475–483. 10.1016/S0166-2236(00)01633-7 11006464

[B17] Elliott R. Newman J. L. Longe O. A. Deakin J. F. (2003). Differential response patterns in the striatum and orbitofrontal cortex to financial reward in humans: a parametric functional magnetic resonance imaging study. J. Neurosci. 23, 303–307. 12514228 10.1523/JNEUROSCI.23-01-00303.2003 PMC6742125

[B18] Ernst M. Paulus M. P. (2005). Neurobiology of decision making: a selective review from a neurocognitive and clinical perspective. Biol. Psychiatry 58, 597–604. 10.1016/j.biopsych.2005.06.004 16095567

[B19] Foerde K. Shohamy D. (2011). The role of the basal ganglia in learning and memory: insight from Parkinson's disease. Neurobiol. Learn. Mem. 96, 624–636. 10.1016/j.nlm.2011.08.006 21945835 PMC3772079

[B20] Fukunaga R. Brown J. W. Bogg T. (2012). Decision making in the Balloon Analogue Risk Task (BART): anterior cingulate cortex signals loss aversion but not the infrequency of risky choices. Cogn. Affect. Behav. Neurosci. 12, 479–490. 10.3758/s13415-012-0102-1 22707378 PMC3493559

[B21] Grahn J. A. Parkinson J. A. Owen A. M. (2009). The role of the basal ganglia in learning and memory: neuropsychological studies. Behav. Brain Res. 199, 53–60. 10.1016/j.bbr.2008.11.020 19059285

[B22] Greene J. D. Sommerville R. B. Nystrom L. E. Darley J. M. Cohen J. D. (2001). An fMRI investigation of emotional engagement in moral judgment. Science 293, 2105–2108. 10.1126/science.1062872 11557895

[B23] Hecht D. Walsh V. Lavidor M. (2010). Transcranial direct current stimulation facilitates decision making in a probabilistic guessing task. J. Neurosci. 30, 4241–4245. 10.1523/JNEUROSCI.2924-09.2010 20335459 PMC6634503

[B24] Holroyd C. B. Coles M. G. (2002). The neural basis of human error processing: reinforcement learning, dopamine, and the error-related negativity. Psychol. Rev. 109, 679–709. 10.1037/0033-295X.109.4.679 12374324

[B25] Hsu M. Bhatt M. Adolphs R. Tranel D. Camerer C. F. (2005). Neural systems responding to degrees of uncertainty in human decision-making. Science 310, 1680–1683. 10.1126/science.1115327 16339445

[B26] Huettel S. A. Stowe C. J. Gordon E. M. Warner B. T. Platt M. L. (2006). Neural signatures of economic preferences for risk and ambiguity. Neuron 49, 765–775. 10.1016/j.neuron.2006.01.024 16504951

[B27] Knoch D. Fehr E. (2007). Resisting the power of temptations: the right prefrontal cortex and self-control. Ann. N. Y. Acad. Sci. 1104, 123–134. 10.1196/annals.1390.004 17344543

[B28] Krishnan-Sarin S. Balodis I. M. Kober H. Worhunsky P. D. Liss T. Xu J. S.. (2013). An exploratory pilot study of the relationship between neural correlates of cognitive control and reduction in cigarette use among treatment-seeking adolescent smokers. Psychol. Addict. Behav. 27, 526–532. 10.1037/a0032479 23586458 PMC4106014

[B29] Kuhnen C. M. Knutson B. (2005). The neural basis of financial risk taking. Neuron 47, 763–770. 10.1016/j.neuron.2005.08.008 16129404

[B30] Lecrubier Y. Sheehan D. V. Weiller E. Amorim P. Bonora I. Harnett Sheehan K. (1997). The Mini International Neuropsychiatric Interview (MINI). A short diagnostic structured interview: reliability and validity according to the CIDI. Eur. Psychiatry 12, 224–231.

[B31] Lewis A. H. Porcelli A. J. Delgado M. R. (2014). The effects of acute stress exposure on striatal activity during Pavlovian conditioning with monetary gains and losses. Front. Behav. Neurosci. 8:179. 10.3389/fnbeh.2014.00179 24904331 PMC4033231

[B32] Pabst S. Brand M. Wolf O. T. (2013). Stress effects on framed decisions: there are differences for gains and losses. Front. Behav. Neurosci. 7:142. 10.3389/fnbeh.2013.00142 24130523 PMC3793125

[B33] Paulus M. P. Rogalsky C. Simmons A. Feinstein J. S. Stein M. B. (2003). Increased activation in the right insula during risk-taking decision making is related to harm avoidance and neuroticism. Neuroimage 19, 1439–1448. 10.1016/S1053-8119(03)00251-9 12948701

[B34] Platt M. L. Huettel S. A. (2008). Risky business: the neuroeconomics of decision making under uncertainty. Nat. Neurosci. 11, 398–403. 10.1038/nn2062 18368046 PMC3065064

[B35] Plewnia C. Bischof F. Reimold M. (2007). Suppression of verbal hallucinations and changes in regional cerebral blood flow after intravenous lidocaine: a case report. Prog. Neuropsychopharmacol. Biol. Psychiatry 31, 301–303. 10.1016/j.pnpbp.2006.08.014 17011097

[B36] Radua J. Phillips M. L. Russell T. Lawrence N. Marshall N. Kalidindi S.. (2010). Neural response to specific components of fearful faces in healthy and schizophrenic adults. Neuroimage 49, 939–946. 10.1016/j.neuroimage.2009.08.030 19699306

[B37] Remijnse P. L. van den Heuvel O. A. Nielen M. M. A. Vriend C. Hendriks G.-J. Hoogendijk W. J. G.. (2013). Cognitive inflexibility in obsessive-compulsive disorder and major depression is associated with distinct neural correlates. PLoS ONE 8:e59600. 10.1371/journal.pone.0059600 23637737 PMC3634812

[B38] Rothman A. J. Salovey P. (1997). Shaping perceptions to motivate healthy behavior: the role of message framing. Psychol. Bull. 121, 3–19. 10.1037/0033-2909.121.1.3 9000890

[B39] Rushworth M. F. Kolling N. Sallet J. Mars R. B. (2012). Valuation and decision-making in frontal cortex: one or many serial or parallel systems? Curr. Opin. Neurobiol. 22, 946–955. 10.1016/j.conb.2012.04.011 22572389

[B40] San Martín R. Appelbaum L. G. Huettel S. A. Woldorff M. G. (2014). Cortical brain activity reflecting attentional biasing toward reward-predicting cues covaries with economic decision-making performance. Cereb. Cortex. 10.1093/cercor/bhu160. [Epub ahead of print]. 25139941 PMC4677969

[B41] Schonberg T. Fox C. R. Poldrack R. A. (2011). Mind the gap: bridging economic and naturalistic risk-taking with cognitive neuroscience. Trends Cogn. Sci. 15, 11–19. 10.1016/j.tics.2010.10.002 21130018 PMC3014440

[B42] Seger C. A. Cincotta C. M. (2005). The roles of the caudate nucleus in human classification learning. J. Neurosci. 25, 2941–2951. 10.1523/JNEUROSCI.3401-04.2005 15772354 PMC6725143

[B43] Sheth S. A. Mian M. K. Patel S. R. Asaad W. F. Williams Z. M. Dougherty D. D.. (2012). Human dorsal anterior cingulate cortex neurons mediate ongoing behavioural adaptation. Nature 488, 218–221. 10.1038/nature11239 22722841 PMC3416924

[B44] Shiv B. Loewenstein G. Bechara A. (2005). The dark side of emotion in decision-making: when individuals with decreased emotional reactions make more advantageous decisions. Brain Res. Cogn. Brain Res. 23, 85–92. 10.1016/j.cogbrainres.2005.01.006 15795136

[B45] Tom S. M. Fox C. R. Trepel C. Poldrack R. A. (2007). The neural basis of loss aversion in decision-making under risk. Science 315, 515–518. 10.1126/science.1134239 17255512

[B46] Tricomi E. Delgado M. R. McCandliss B. D. McClelland J. L. Fiez J. A. (2006). Performance feedback drives caudate activation in a phonological learning task. J. Cogn. Neurosci. 18, 1029–1043. 10.1162/jocn.2006.18.6.1029 16839308

[B47] Tricomi E. Fiez J. A. (2008). Feedback signals in the caudate reflect goal achievement on a declarative memory task. Neuroimage 41, 1154–1167. 10.1016/j.neuroimage.2008.02.066 18445531 PMC2487673

[B48] van Veen V. Holroyd C. B. Cohen J. D. Stenger V. A. Carter C. S. (2004). Errors without conflict: implications for performance monitoring theories of anterior cingulate cortex. Brain Cogn. 56, 267–276. 10.1016/j.bandc.2004.06.007 15518940

[B49] Venkatraman V. Payne J. W. Bettman J. R. Luce M. F. Huettel S. A. (2009). Separate neural mechanisms underlie choices and strategic preferences in risky decision making. Neuron 62, 593–602. 10.1016/j.neuron.2009.04.007 19477159 PMC3213208

[B50] Xue G. Lu Z. Levin I. P. Bechara A. (2010). The impact of prior risk experiences on subsequent risky decision-making: the role of the insula. Neuroimage 50, 709–716. 10.1016/j.neuroimage.2009.12.097 20045470 PMC2828040

[B51] Xue G. Lu Z. Levin I. P. Bechara A. (2011). An fMRI study of risk-taking following wins and losses: implications for the gambler's fallacy. Hum. Brain Mapp. 32, 271–281. 10.1002/hbm.21015 21229615 PMC3429350

